# Three-year outcomes of a fracture liaison service model at a university-based tertiary care hospital in Thailand

**DOI:** 10.1007/s11657-023-01215-z

**Published:** 2023-01-24

**Authors:** Pojchong Chotiyarnwong, Nitchanant Kitcharanant, Ekasame Vanitcharoenkul, Chirathit Anusitviwat, Atthakorn Jarusriwanna, Worasit Suthutvoravut, Wararat Boonnasa, Aasis Unnanuntana

**Affiliations:** 1 Department of Orthopaedic Surgery, Faculty of Medicine Siriraj Hospital, Mahidol University, 2 Wanglang Road, Bangkok Noi, Bangkok, 10700 Thailand; 2 Department of Orthopedics, Faculty of Medicine, Chiang Mai University, Chiang Mai, Thailand; 3 Department of Orthopedics, Faculty of Medicine, Prince of Songkla University, Songkhla, Thailand; 4 Department of Orthopaedics, Faculty of Medicine, Naresuan University, Phitsanulok, Thailand; 5 Department of Orthopedics, Bhumibol Adulyadej Hospital, Bangkok, Thailand; 6 Department of Nursing, Faculty of Medicine Siriraj Hospital, Mahidol University, Bangkok, Thailand

**Keywords:** Anti-osteoporosis, Fracture liaison service (FLS), Fragility hip fracture (FHF), Functional outcomes, Mortality rate, Refracture rate

## Abstract

***Summary*:**

Fragility hip fracture (FHF) is a serious complication of osteoporosis. A fracture liaison service (FLS) is crucial in preventing FHF. Our retrospective data of 489 patients with FHF and 3-year follow-ups demonstrated that the FLS improved functional outcomes. Our study’s mortality rates were lower than in other published series.

**Purpose:**

This study assessed the 3-year outcomes after fragility hip fracture (FHF) treatment by a multidisciplinary team from the Siriraj Fracture Liaison Service (Si–FLS). The review investigated the administration rates of anti-osteoporosis medication, refracture, and mortality; activities of daily living; mobility; and health-related quality of life.

**Methods:**

A retrospective review was performed of the records of Si-FLS patients given FHF treatment between June 2016 and October 2018. The outcomes were evaluated at 3 time points: before discharge, and 1 and 3 years after treatment.

**Results:**

The study enrolled 489 patients (average age, 78). The mortality and refracture rates at 1 year after hip fracture were 13.9% and 1.6%, respectively. At the 3-year follow-up, both rates were higher (20.4% and 5.7%, respectively). The Barthel Index and EuroQoL Visual Analogue Scale had risen to a plateau at the 1-year follow-up and remained stable to the 3-year follow-up. One year after treatment, approximately 60% of the patients could ambulate outdoors, and the proportion remained steady until the 3-year follow-up. There was no difference in the 1- and 3-year follow-up anti-osteoporosis medication administration rates (approximately 40%).

**Conclusions:**

This study confirms the benefits of having a multidisciplinary FLS care team to manage older people with FHF. An FLS improves the care of patients with FHF and the social support of caregivers and relatives. The FLS maintained the functional outcomes of the patients through 3 years of postfracture treatment.

## Introduction 

Fragility hip fracture (FHF) is one of the most severe complications of osteoporosis [1]. Without appropriate treatment, there is a high risk of a subsequent fragility fracture, which causes significant morbidity and mortality [2]. Although assessment and treatment for osteoporosis are of prime importance in preventing secondary fracture, their adequate provision is an issue worldwide [3]. In Asia, only one-third of patients with an FHF receive osteoporosis treatment [4].

Concerted efforts to improve the diagnosis, treatment, and prevention of FHFs are necessary to mitigate their ongoing strain on national economies and societies. Several programs have therefore been developed, such as the “Own the Bone” [5] and “Capture the Fracture” [6] initiatives. These aim to raise physicians’ awareness of FHF treatments, promote optimum treatment and care plans, and encourage long-term follow-up of patients with FHFs. The programs typically use a strategic secondary-fracture prevention model now commonly known as a fracture liaison service (FLS). The FLS is a cost-effective measure that can reduce the rate of secondary fragility fractures, increase the rate of osteoporosis treatment, and improve the quality of patient care [7].

Few studies on FLS have been conducted in Thailand. In addition, some core clinical outcomes are underreported, such as postinjury mobility, performance in activities of daily living, and quality of life after using an FLS [8]. Therefore, this study aimed to report the 3-year outcomes of the FLS at our institution using various outcome measures. They were mortality rate, the proportion of patients who sustained a recurrent fracture, activities of daily living, and patients’ health-related quality of life. The results of this study reflect the real-world effectiveness of an FLS model in a university-based tertiary care hospital setting.

## Methods

Before this research began, the Institutional Review Board approved its protocol (COA no. Si 754/2019). We retrospectively reviewed patient data recorded in the Siriraj–FLS Registry between July 2016 and October 2018. The investigation only assessed patients who had been diagnosed with femoral neck and intertrochanteric fractures and who had a minimum follow-up of 1 year or until death. The exclusion criteria were as follows:

- Patients diagnosed with a pathological fracture confirmed by a pathological study. These patients were excluded since their prognoses differ from those of patients with an osteoporotic fracture.
- Patients who sustained multiple injuries or fractures. This group of patients was excluded because their rehabilitation programs and recovery are different from those of patients sustaining a hip fracture only.

### Siriraj Fracture Liaison Service

A multidisciplinary care team assessed all patients with an FHF and treated them according to our center’s hip fracture protocol [9]. After the treatment, video-based osteoporosis education and supplementary reading material were provided to the patients and their caregivers. A metabolic bone disease specialist team reviewed each patient’s profile, and an appropriate anti-osteoporosis medication was suggested. Vitamin D2 supplementation was prescribed according to patients’ baseline vitamin D levels as previously described [10]. The dosage was 60,000 IU/week when patients’ baseline vitamin D levels were below 20 ng/mL, 40,000 IU/week for levels between 20 and 30 ng/mL, 20,000 IU/week for levels between 30 and 40 ng/mL, and zero for levels exceeding 40 ng/mL. A fall-prevention protocol, including any necessary home modifications, was developed by the multidisciplinary care team. Once a patient was deemed fit for discharge, an FLS nurse coordinator transferred the postoperative care plan to the treating physicians. Patients and caregivers were provided basic exercise and home physical therapy information.

### Assessment of outcomes

Demographic data and clinical information were collected as follows: age, sex, body mass index, Charlson Comorbidity Index, the percentage of 10-year probability of fracture by the fracture risk assessment tool (FRAX) [11], history of fractures, preinjury ambulatory status, fracture site, and treatment type. Our outcomes of interest were the following:

- mortality rate
- refracture rate (defined as any clinical osteoporotic fracture)
- the proportion of patients receiving calcium and vitamin D supplementation
- the proportion of patients given anti-osteoporosis medications

In addition, we determined the bone mineral density (BMD) assessment rate during the first year after FHF treatment. We also collected details of functional outcome measures as follows:

- activities of daily living (using the Barthel Index)
- health-related quality of life (using the EuroQoL–Visual Analogue Scale)
- postfracture ambulatory status

Patients’ postfracture ambulatory statuses were classified as bedridden, indoor ambulator, and outdoor ambulator. All outcome measures were evaluated at 3 time points: before discharge, and 1 and 3 years after treatment. The postdischarge evaluations were conducted by telephone interviews with the patients or, if they could not communicate via telephone, their primary caregivers.

#### Barthel Index

The Barthel Index (BI) is a 10-item ordinal scale used to evaluate patients’ functional independence in performing their activities of daily living. Mahoney and Barthel DW introduced the BI in 1965 [12]. It has a total possible score of 100, with higher scores indicating high degrees of mobility in the activities of daily living. A Thai-language version of the scoring system has been validated for use with older patients with FHFs [13].

#### EuroQoL-Visual Analogue Scale

The EuroQoL-Visual Analogue Scale (EQ-VAS) records a patient’s self-rated health status on a vertical 20-cm visual analog scale. Its grading ranges from “0” (the worst possible health status that you can imagine) to “100” (the best possible health status that you can imagine). Patients mark an “X” on the scale in the position that reflects their perception of their current health status. This tool has been validated in older adults with FHFs [9, 14].

### Statistical analysis

Descriptive statistics were used. Continuous variables are presented as the means, standard deviations, and ranges, while categorical variables are summarized as frequencies and percentages. Changes in BI and EQ-VAS scores from discharge to 1 and 3 years after a hip fracture were assessed with a one-way repeated-measure analysis of variance (one-way repeated ANOVA). The BI and EQ-VAS scores at discharge and the 1- and 3-year follow-ups were compared using post hoc analysis with Bonferroni correction. Data analyses were performed using PASW Statistics for Windows, version 18 (SPSS Inc., Chicago, IL, USA). Probability (*P*) values ≤ 0.05 were considered statistically significant.

## Results

From July 2016 to October 2018, data on 489 patients with FHFs were entered into the Siriraj–FLS Registry. Their average age was 78.4 years, and most were female (72%). The average body mass index was approximately 22.3 kg/m^2^, and over 90% had a Charlson Comorbidity Index ≥ 3. Equal proportions of patients were diagnosed with femoral neck and intertrochanteric femoral fractures. Most patients received surgical treatment for FHF. Nearly 60% of the patients were outdoor ambulators before their injury (Table 1).

**Table 1:** Patient demographic and clinical characteristics

Characteristics	Hip fracture patients (*N* = 489)
Female sex	352 (72.0%)
Age (years)	78.4 ± 9.8
Body mass index (kg/m^2^)	22.3 ± 3.9
Charlson Comorbidity Index	
< 3	36 (7.3%)
≥ 3	453 (92.6%)
History of previous fracture	15 (3.1%)
Preinjury ambulatory status	
Bedridden	15 (3.1%)
Indoor ambulator	188 (38.5%)
Outdoor ambulator	286 (58.5%)
Site of fracture	
Femoral neck	248 (50.7%)
Intertrochanter	241 (49.3%)
Type of hip fracture treatment	
Conservative	32 (6.5%)
Fixation	254 (52.0%)
Arthroplasty	203 (41.5%)

**Fig. 1:**
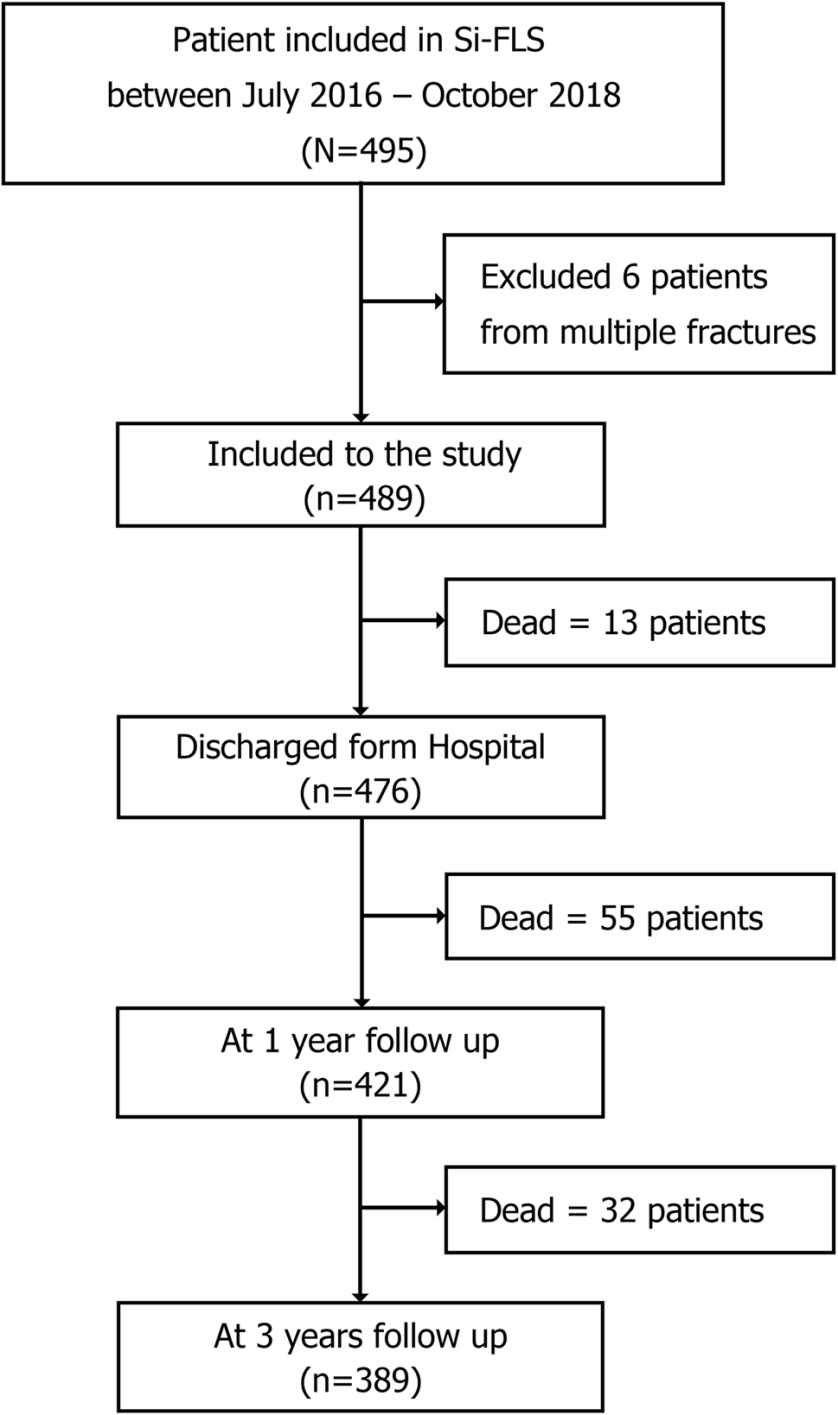
The flow of patients managed by the Siriraj–FLS from their admission through their 3-year follow-up, between July 2016 and October 2018

During the 3-year follow-up, the cumulative deaths were 13, 68, and 100 at discharge and 1 and 3 years after hip fracture treatment, respectively. Thus, the in-hospital mortality rate of our patient population was 2.7%; the rate rose to 13.9% and 20.4% at 1 and 3 years after hip fracture treatment, respectively. During the data collection period, excluding deaths, none of our patients was lost to follow-up. Therefore, the total numbers of patients available for statistical analysis were 476, 421, and 389 at discharge and the 1- and 3-year follow-ups, respectively (Fig. 1).

During hospitalization, all patients and their caregivers received video-based osteoporosis educational material. BMD assessments were carried out on 397 patients (81.2%) 1 year after their FHFs. The proportion of patients who received calcium and vitamin D supplementation was 98.3% at discharge, and this proportion remained at approximately 90% during the 3-year follow-up. Regarding anti-osteoporosis medications, the rate of prescribing anti-osteoporosis medication before discharge was only 13.4%. At the 1- and 3-year follow-ups, the proportion of patients using anti-osteoporosis medication had risen to approximately 40% (Table 2). Oral bisphosphonates were the most commonly prescribed anti-osteoporosis agents. Interestingly, of those who received anti-osteoporosis medication within 1 year after their hip fracture, only 13% had not received BMD testing, while 87% had DXA results (*P* < 0.001).

**Table 2:** Key indices of patients at discharge and at the 1- and 3-year follow-ups

Key performance indices	Discharge (*n* = 476)	1-year post-fracture (*n* = 421)	3-year post-fracture (*n* = 389)
Rate of BMD assessment*		397 (81.2%)	
Rate of calcium and vitamin D supplementation	468 (98.3%)	391 (92.8%)	352 (90.5%)
Rate of treatment with anti-osteoporosis medication	64 (13.4%)	171 (40.6%)	153 (39.3%)
Oral bisphosphonate	37 (7.8%)	114 (27.1%)	98 (25.2%)
Intravenous bisphosphonate	7 (1.5%)	10 (2.4%)	6 (1.5%)
Denosumab	13 (2.7%)	42 (10.0%)	44 (11.3%)
Teriparatide	5 (1.1%)	4 (1.0%)	5 (1.3%)
Strontium ranelate**	2 (0.4%)	1 (0.2%)	0 (0%)
Post-fracture ambulatory status			
Bedridden	47 (9.9%)	47 (11.2%)	34 (8.7%)
Indoor ambulator	364 (76.5%)	101 (24.0%)	129 (33.2%)
Outdoor ambulator	65 (13.7%)	273 (64.8%)	226 (58.1%)
Rate of refracture*	0 (0.0%)	8 (1.6%)	28 (5.7%)

^*^The rates were calculated based on the total of 489 FHF patients enrolled in this study

^**^Strontium ranelate has not been available since 2017

Regarding postfracture ambulatory status, the proportions of bedridden patients were 9.9%, 11.2%, and 8.7% at discharge and at the 1- and 3-year follow-ups, respectively. The rate of patients who could ambulate outdoors at discharge was only 13.7%. However, the proportion rose substantially to approximately 65% and 58% at the 1- and 3-year follow-ups, respectively (Table 2). Eight patients (1.6%) sustained a secondary fracture within 1 year of their hip fracture treatment. At the minimum follow-up of 3 years, the rate of subsequent fractures was 5.7%. The 3 most common sites of recurrent fractures were the contralateral hip (3.9%), the distal femur (0.6%), and the distal radius (0.4%). The average 10-year probability of a major osteoporotic fracture was 13.2 ± 6.8%, while the 10-year probability of a hip fracture was 6.8 ± 5.2%.

**Fig. 2:**
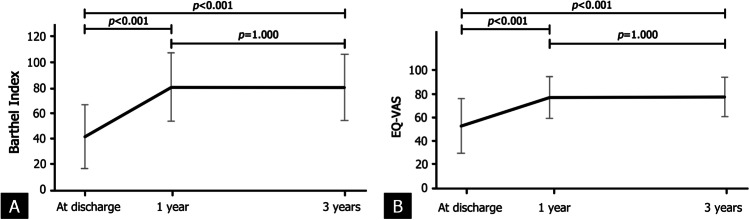
Overall mean Barthel Index (**A**) and EQ-VAS (**B**) scores at discharge, and at the 1-year and 3-year follow-ups, of the patients with fragility hip fracture managed by the Siriraj-FLS

Regarding the functional outcomes and quality of life of the patients, the mean and standard deviation of the BI score at discharge was 42.1 ± 25.0. This score increased significantly, reaching 80.6 ± 26.6 at the 1-year follow-up (*P* < 0.001) and remained stable (*P* = 1.000) until the 3-year follow-up (80.6 ± 25.7; Fig. 2A). Similarly, the mean baseline EQ-VAS score at discharge (52.7 ± 23.1) improved significantly to a plateau of 77.0 ± 17.5 at the 1-year follow-up. The score then remained steady to the 3-year follow-up assessment (77.4 ± 16.6; Fig. 2B).

## Discussion

With the populations in many countries around the world aging rapidly, higher incidences of fragility fractures are inevitable. These fractures are associated with increased disability, morbidity, and mortality. If these problems are not satisfactorily addressed, the rise in fragility fractures will adversely impact patients’ health and place substantial economic burdens on societies [15–18]. Our outcomes support that an FLS can be beneficially used to care for patients with fragility fractures. As at September 2022, 17 FLSs in Thailand were included in the “Map of Best Practice” maintained by the International Osteoporosis Foundation (https://www.capturethefracture.org/map-of-best-practice).

Compared with other reports from the Asia–Pacific region [8], a substantial proportion of the patients in our study cohort (81.2%) underwent BMD assessment 1 year after their hip fractures. This rate of BMD testing was much higher than the median rate reported by a systematic review and meta-analysis (81.2% vs 48%) [19]. Of the patients who underwent BMD assessments, the proportion who received anti-osteoporosis medications was significantly higher than the proportion who did not (87% vs 13%). In 2019, Kittithamvongs and Pongpirul reported that BMD results influence physicians’ decisions regarding the prescription of anti-osteoporosis medications to patients with osteoporotic hip fractures [20]. Additionally, in a country neighboring Thailand, inaccessibility to BMD testing was found to be a barrier to osteoporosis management [21]. Therefore, our results suggest that increasing the BMD assessment rate could raise awareness of the need for anti-osteoporosis medication prescriptions.

In addition, the refracture rate of our Siriraj-FLS patients (5.7% at the 3-year follow-up) was considered low and comparable to those reported in a systematic review (0–6.5%) [8] and a meta-analysis (6.4%) [19]. Drawing upon the 10-year fracture probabilities calculated by FRAX, our 10-year probabilities of major osteoporotic and hip fractures were 13.2 ± 6.8% and 6.8 ± 5.2%, respectively. The observed percentage of (new) fractures after 3 years of the index hip fracture was still under the estimated percentage of fractures at 10 years. It would be interesting to monitor these patients over an extended follow-up period to assess the validity of the FRAX for Thais.

Our low refracture rate might result from several factors. One is our comprehensive fall prevention program, which is part of the osteoporosis education given to all patients and their caregivers. The other factor is the high rate of BMD testing, which facilitates the prevention of secondary fragility fractures by identifying patients at risk and encouraging their compliance with anti-osteoporosis therapy. It is also important to note that over 90% of our patients received calcium and vitamin D supplementation. As for those who did not receive supplementation, most had serum calcium and vitamin D levels within the normal ranges.

The mean 1-year BI and EQ-VAS scores increased dramatically from their baseline values. Afterwards, there were no statistically significant differences between our BI and EQ-VAS scores at the 1-year follow-up and their corresponding values at the 3-year follow-up (*P* = 1.000 for both EQ-VAS and BI). Our mean post-hip fracture BI and EQ-VAS scores are comparable to the scores reported by previous studies. For instance, Chiang et al. [22] and Imai et al. [23] reported mean BI scores of 71.1 and 71.9 points at 1- and 2-year post-hip fracture treatment, respectively. Similarly, our mean 1-year EQ-VAS score (77.0 ± 17.5) is comparable to the mean of 67 ± 2 reported by Svedbom et al. [24] and the mean of 80 ± 10 found by van der Vet et al. [25]

Our 1- and 3-year mortality rates were 13.9% and 20.4%, respectively. These numbers were compatible with those reported in a previous systematic review and meta-analysis of various centers with an FLS care model [8, 19]. In contrast, Vaseenon et al. reported a much higher mortality rate in Thai patients with FHF who were not under the care of an FLS program. Those patients had 1- and 3-year mortality rates of 18% and 32%, respectively [26]. The lower rates for patients under FLS management probably stem from the multidisciplinary team approach with a dedicated nurse coordinator. Having a dedicated nurse coordinator appears to be one of the keys to the success of an FLS [27, 28]. Therefore, these findings underscore the effectiveness of an FLS care model to improve the quality of hip fracture care and reduce mortality in this patient population.

Interestingly, in 2005, only 4% of patients in Thailand received anti-osteoporosis medications after hip fracture in centers without an FLS [29]. Our anti-osteoporosis treatment rate after FHF was approximately 40%. This rate was comparable to the unweighted average of 38% found by the meta-analysis [19]. Factors associated with not receiving anti-osteoporosis medication are multifactorial. Among them are healthcare-cost reimbursement schemes (given that oral bisphosphonate was the only anti-osteoporosis agent whose costs were fully covered for all osteoporosis patients in Thailand), healthcare systems, patients and caregivers’ perceptions of osteoporosis treatment, and physicians and policy makers’ beliefs about the benefits of secondary fracture prevention. Therefore, further study is required to delineate the reasons for not-receiving anti-osteoporosis medication. We acknowledge that there is room for improvement in our anti-osteoporosis treatment rate and that new interventions should be employed to improve the initiation of and adherence to anti-osteoporosis medications.

The strength of this study is that our FLS collected data related to a range of core outcomes. They were pre- and postinjury ambulation, performance in activities of daily living, and quality of life after FLS implementation. These outcomes have tended to be underreported in the literature, yet they are crucial to determining the comprehensive status of patients with FHFs. Nonetheless, there are some limitations to this study. First, our study had a retrospective design; nevertheless, the fact that we collected data from our FLS registry minimized potential biases. Second, this study drew upon data from only one center in Thailand, a high-volume hospital with an experienced FLS team. Consequently, some aspects of our data and findings may not be generalizable to centers that provide a less sophisticated level of care or do not have an FLS.

In conclusion, our findings concur with previous reports that FLSs contribute to significant improvements in the rates of osteoporosis education and calcium and vitamin D supplementation, with resulting satisfactory functional outcomes and excellent BMD and refracture rates. Because FLSs are highly beneficial to patients with fragility fractures, they should be established at all centers. Nevertheless, only approximately 40% of our patients with FHFs adhered to their anti-osteoporosis treatment. Further study is needed to identify the reasons for unsatisfactory anti-osteoporosis compliance, and specific interventions should be explored to increase the rate of anti-osteoporosis initiation.

## Data Availability

The data used in this study are available from the corresponding author upon reasonable request.
